# A Fourier Descriptor of 2D Shapes Based on Multiscale Centroid Contour Distances Used in Object Recognition in Remote Sensing Images

**DOI:** 10.3390/s19030486

**Published:** 2019-01-24

**Authors:** Yan Zheng, Baolong Guo, Zhijie Chen, Cheng Li

**Affiliations:** Institute of Intelligent Control and Image Engineering, Xidian University, Taibai Road, Xi’an 710071, China; yanzheng@stu.xidian.edu.cn (Y.Z.); chenzhijie@stu.xidian.edu.cn (Z.C.); licheng812@stu.xidian.edu.cn (C.L.)

**Keywords:** shape descriptor, object recognition, fourier transform

## Abstract

A shape descriptor is an effective tool for describing the shape feature of an object in remote sensing images. Researchers have put forward a lot of excellent descriptors. The discriminability of some descriptors is very strong in the experiments, but usually their computational cost is large, which makes them unsuitable to be used in practical applications. This paper proposes a new descriptor-FMSCCD (Fourier descriptor based on multiscale centroid contour distance)—which is a frequency domain descriptor based on the CCD (centroid contour distance) method, multiscale description, and Fourier transform. The principle of FMSCCD is simple, and the computational cost is very low. What is commendable is that its discriminability is still strong, and its compatibility with other features is also great. Experiments on three databases demonstrate its strong discriminability and operational efficiency.

## 1. Introduction

**Figure 1:**
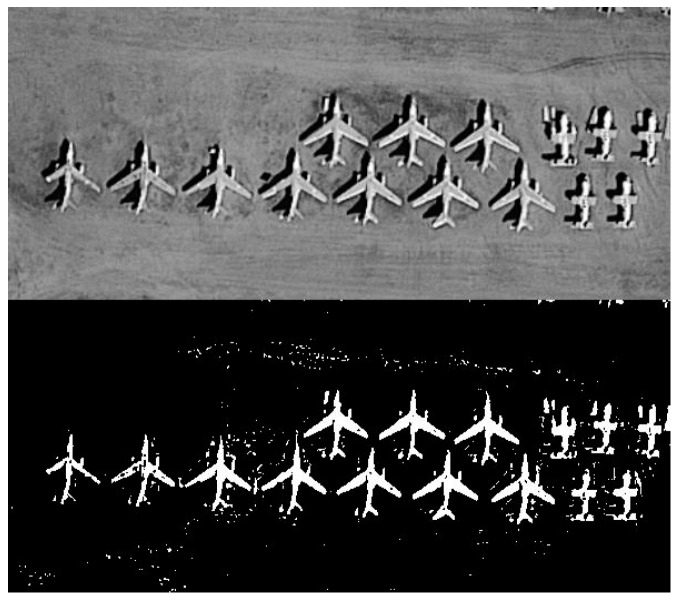
The upper half is a blurred remote sensing image and the lower half shows the shapes of the objects in the upper half.

The objects in remote sensing images are more blurred compared to in common images, therefore, it is hard for recognition with texture and point features, as Figure 1 shows. Without texture features and feature points, people can only use shape features to identify objects. A shape descriptor is a great tool for the task of identifying objects relying on shape features. In addition, theoretically, deep learning can complete object recognition in remote sensing images but establishing a training dataset is a very difficult task since such images are not easy to obtain. Therefore, for object recognition in remote sensing images, a shape descriptor is a suitable tool.

A shape descriptor is always used to extract the shape features of an object in an image. Research on shape descriptors has attracted scholars for more than 20 years. In the past two decades a lot of effective descriptors [1,2,3,4] and post-processing methods [5,6,7] based on machine learning appeared. Among them, IDSC + DP (inner-distance shape context and dynamic programming) [8,9], SC + DP (shape context and dynamic programming) [10], Shape Tree [4], TAR (triangle-area representation) [1], FD (Fourier descriptor) [11], and WD (wavelet descriptor) [12] are some classical descriptors. Co-transduction (Co-transduction for shape retrieval) [13], LCDP (locally constrained diffusion process) [14], and GMM (modified mutual graph) [15] are post-processing methods that are popular recently, however, their high accuracy is still based on the performance of classical descriptors.

FD [11] is a kind of frequency domain descriptor with high practical value because of its excellent balance between speed and precision. FD usually obtains a feature vector in the spatial domain first, then the spatial domain feature vector; for example, CCD (centroid contour distance) is transformed into a frequency domain feature vector. In [11], the FD-CCD (FD based on CCD) obtains the best experimental results on the MPEG-7 CE1 Part B shape database among many combinations. MDM (multiscale distance matrix) [16] is also a descriptor that is known for speed. It uses a multiscale description method to compute a feature matrix for a shape. In the matching process, the dissimilarity is the city block distance between two feature matrices. DIR (distance interior ratio) [17] is a relatively new fast descriptor. It is as fast as FD-CCD, but it is more accurate than FD-CCD.

ASD&CCD [18] is a descriptor which combines the CCD method with the ASD (angle scale descriptor) method. It is more accurate than FD-CCD and MDM, however it runs slowly as it uses an optimization algorithm to find the best correspondence for the starting point. In this paper, Fourier transform and multiscale description are used to improve ASD and CCD.

The ASD [18] feature contains some angle sequences, which are computed at different scales. The element in the sequence is an angle that is formed by a contour point and two other contour points at its front and rear. The two contour points at the front and rear have the same length of interval to the contour point in the middle. The length of interval is how many contour points there are, and it refers to the scale. To improve ASD, each angle sequence is transformed to the frequency domain to form FASD (Fourier descriptor based on ASD).

The CCD [18] feature is a distance sequence. The element in the sequence is the distance between a contour point and the centroid point of the contour. As the CCD method is too simple, improving CCD is the most important work in this article. Fourier transform and multiscale description are all used to form FMSCCD (Fourier descriptor based on multiscale CCD).

IDSC + DP [8] is undoubtedly a great descriptor. It has obtained very high accuracy in experimental results in some databases, but as it uses dynamic programming in the matching process, the matching efficiency is extremely low, which makes it useless in engineering. Shape Tree [4] and TAR [1] are all the same as IDSC + DP, which is accurate but slow. Some researchers also use skeletons to describe shapes [19,20], but skeleton-based methods are less popular than contour-based methods.

Some descriptors based on matching learning [21,22] have appeared in recent years. Sometimes these methods are used in medical images analysis [23]. However, as the training datasets are not easy to obtain, these methods are not universal.

In the remainder of this paper, Section 2 of this paper describes the specific calculation process of the proposed method. In Section 3, some databases are used to evaluate the performance of the proposed method. Section 4 discusses the performance of the proposed method. Finally, this paper is concluded.

## 2. Methods

CCD [18] is a commonly used spatial domain feature. For a sequence $\left\{p_{1},p_{2},\ldots ,p_{N_{p}}\right\}$ of uniform contour points in order, where $N_{p}$ means how many sampling points there are on the contour, the centroid contour point of these contour points is first calculated, using Equation (1).

(1) $$p_{m}=\frac{1}{N_{p}}\sum_{i=1}^{N_{p}}p_{i}=(\frac{1}{N_{p}}\sum_{i=1}^{N_{p}}x_{i},\frac{1}{N_{p}}\sum_{i=1}^{N_{p}}y_{i},),$$

where $p_{i}=(x_{i},y_{i})$ is the ith contour point of a shape. Then, the Euclidean distance between each contour point and the centroid contour point $p_{m}=(x_{m},y_{m})$ is calculated, using Equation (2).

(2) $$d_{uc}^{i}={\left({\left(x_{i}-x_{m}\right)}^{2}+{\left(y_{i}-y_{m}\right)}^{2}\right)}^{1/2},i=1,2,\ldots ,N_{p},$$

where $d_{uc}^{i}$ is the unnormalized Euclidean distance between the ith point and the centroid contour point $p_{m}$. $d_{ccd}^{i}$ is the normalized distance, centroid contour distance, calculated with Equation (3). The purpose of normalization is to make the feature scaling invariant and reduce the disturbance caused by the number of sampling points changing.

(3) $$d_{ccd}^{i}=(d_{uc}^{i}/(\frac{1}{N_{p}}\sum_{j=1}^{N_{p}}d_{uc}^{j}))/\sqrt{N_{p}}=d_{uc}^{i}\sqrt{N_{p}}/\sum_{j=1}^{N_{p}}d_{uc}^{j},i=1,2,\ldots ,N_{p}$$

The sequence $D_{ccd}=\{d_{ccd}^{1},d_{ccd}^{2},\ldots ,d_{ccd}^{N_{p}}\}$ is the CCD feature of a shape.

The CCD feature can be used directly to describe a shape. However, there is a problem in the CCD method. When the starting position of the sampling point of the closed contour changes, the CCD feature vector will cyclically shift. Therefore, in the CCD feature space, the distance between two shapes, $s_{1}$ and $s_{2}$, is computed with Equation (4).

(4) $$dis_{ccd}(s_{1},s_{2})=\underset{0\leq n<N_{p}}{\min}\;{\left(\sum_{i=1}^{N_{p}}{\left(d_{ccd,s_{1}}^{i}-d_{ccd,s_{2}}^{n+i}\right)}^{2}\right)}^{1/2},n\in Z,$$

where $dis_{ccd}(s_{1},s_{2})$ is the distance/dissimilarity between two shapes $s_{1}$ and $s_{2}$ in the CCD feature space and $d_{ccd}^{n+i}=d_{ccd}^{n+i-N_{p}}$ exists as the contour is closed.

The optimization problem shown in Equation (4) is a non-convex optimization, so the general convex optimization solution methods are not applicable. Evolutionary algorithms can be used for solving, but with lower efficiency. Therefore, the CCD method has no advantage in efficiency. Some scholars use Fourier transform [7] to transform CCD into a frequency domain feature, FD-CCD (Fourier descriptor based on CCD), which was obtained with Equation (5).

(5) $$F_{ccd}(k)=\frac{1}{N_{p}}\left|\sum_{i=0}^{N_{p}-1}d_{ccd}^{i}e^{\frac{-j2\pi ik}{N_{p}}}\right|,k=0,1,\ldots ,N_{p}-1$$

The matching method of $F_{ccd}$ is based on the city block distance, shown as Equation (6).

(6) $$dis_{fc}(s_{1},s_{2})=\sum_{k=0}^{K}\left|F_{ccd}^{s_{1}}(k)-F_{ccd}^{s_{2}}(k)\right|,0\leq K\leq N_{p},$$

where $s_{1}$ and $s_{2}$ are the index numbers of two shapes and K means how many coefficients of the frequency domain feature are used in the matching process. In this article, K=50 exists.

The ASD [18] feature also can be transformed into the frequency domain feature FASD through the Fourier transform in the same way, thereby improving the efficiency in the matching stage. The FASD feature is used in the experimental part. The matching efficiency of FCCD (Fourier descriptor based on centroid contour distance) is greatly improved, but there is not much improvement in terms of accuracy.

In order to demonstrate the discriminability of the CCD method, the exhaustive method is temporarily used in its matching process. Using the CCD method in shape matching in the MPEG-7 CE1 Part B shape database, $dis_{ccd}^{d}$, the average distance between different classes, is 0.3330, calculated with Equation (7).

(7) $$dis_{ccd}^{d}=\sum_{s_{2}=1}^{N_{d}}\sum_{s_{1}=1}^{N_{d}}dis_{ccd}(s_{1},s_{2})(1-sign(s_{1},s_{2})),$$

where the database contains $N_{d}$ shapes. $sign(s_{1},s_{2})$ indicates if two shapes are in the same class, shown in Equation (8).

(8) $$sign(s_{1},s_{2})=\{\begin{array}{c} 1,label(s_{1})=label(s_{2})\\ 0,otherwise \end{array},$$

where label() is the class label of a shape. $dis_{ccd}^{s}$, the average distance between the same class, is 0.1322 calculated with Equation (9).

(9) $$dis_{ccd}^{s}=\sum_{s_{1}=1}^{N_{d}}\sum_{s_{2}=1}^{N_{d}}dis_{ccd}(s_{1},s_{2})sign(s_{1},s_{2})$$

Generally, a threshold $t_{ccd}$ ($dis_{ccd}^{s}<t_{ccd}<dis_{ccd}^{d}$) is set for shape matching. When the distance between two shapes is larger than $t_{ccd}$, they can be determined to be in different classes. When the distance between two shapes is smaller than $t_{ccd}$, they can be determined to be in the same class. This method of judging is slightly rudimentary, but it is of high value in engineering practice.

**Figure 2:**
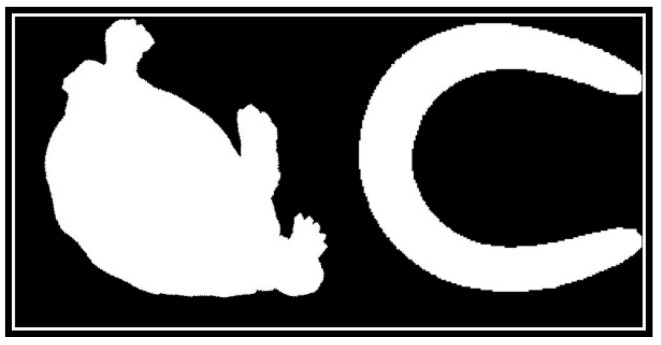
Two shapes that are in different classes in the MPEG-7 CE1 Part B shape database.

**Figure 3:**
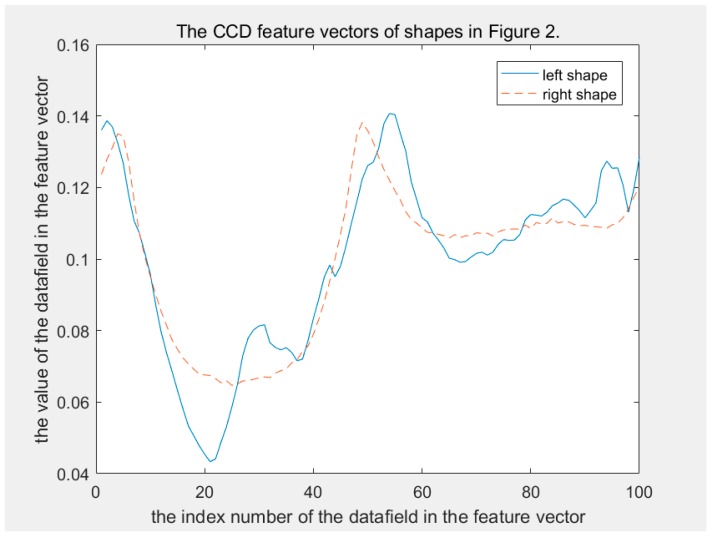
The solid and dashed lines are the centroid contour distance (CCD) feature vectors of the left and right shapes, respectively, in Figure 2. These two curves are similar globally.

**Figure 4:**
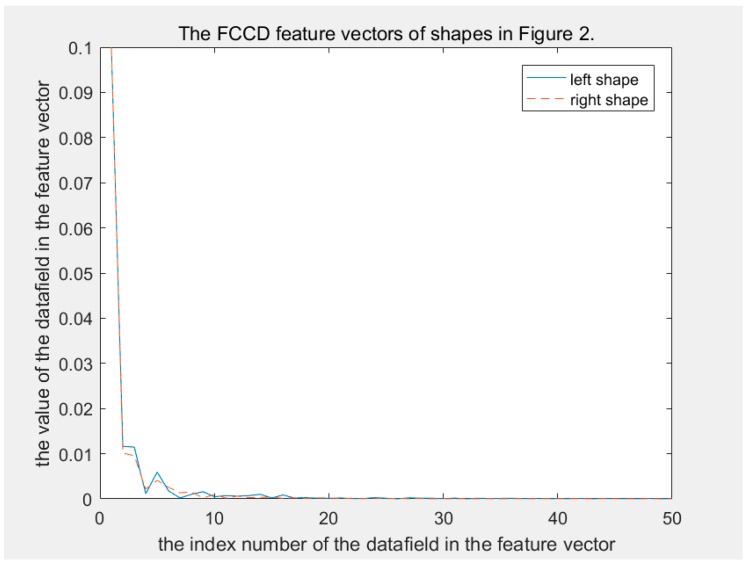
The solid and dashed lines are the FCCD feature vectors of the left and right shapes, respectively, in Figure 2. These two curves are similar, and even a large part of them overlap.

In terms of discriminability, CCD and FCCD have the same weakness, as FCCD is derived from CCD. Human eyes can easily distinguish between two shapes in Figure 2. However, the CCD method does not. In the CCD feature space, the distance between the two shapes in Figure 2 is 0.1007, which is significantly less than $dis_{ccd}^{s}$. This makes them extremely easy to be judged as in the same class. Figure 3 shows their CCD feature vector curves; it can be seen that their feature vectors are so similar. Figure 4 shows their FCCD feature vector curves that are still so similar. What caused this error? This is because $d_{ccd}^{i}$ is a distance scalar without direction. The direction information of the contour points relative to the centroid point is lost during calculation of $d_{ccd}^{i}$, which results in different shapes having similar CCD feature vectors. The FCCD feature is derived from the CCD feature, so it also inherits this error description.

**Figure 5:**
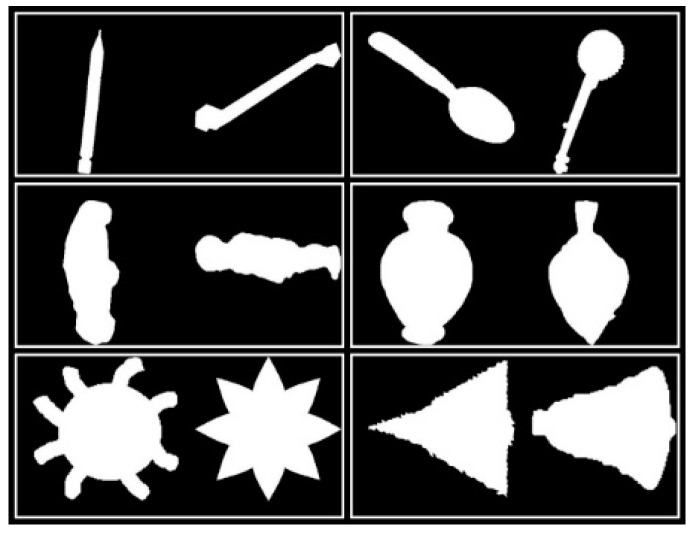
Six pairs of shapes, each pair of which shows two globally similar shapes with only a little detail difference. The first pair in the first row and the first column shows a pencil and a bone. The second pair in the first row and second column shows a spoon and a banjo. The third pair in the second row and the first column shows a car and a person. The fourth pair in the second row and the second column shows a bottle and a fish. The fifth pair in the third row and the first column shows an octopus and an eight-pointed star. The sixth pair in the third row and the second column shows a tree and a bell.

**Figure 6:**
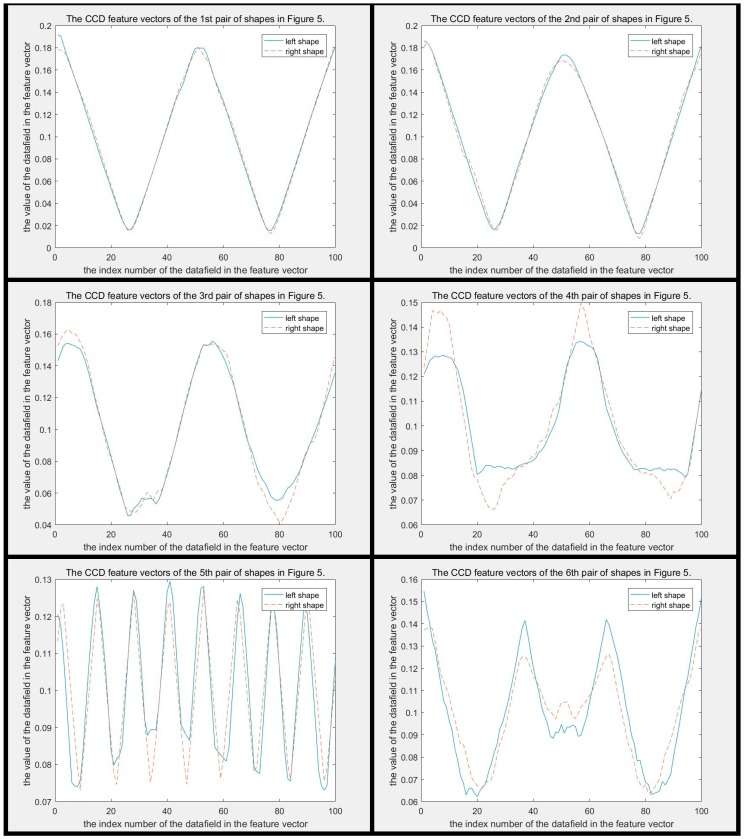
This figure shows the difference between two CCD feature vectors of each pair of shapes in Figure 5. The subfigure of each pair is arranged in the same order as in Figure 5. It can be seen that two curves of each pair are so similar, and even overlap.

**Figure 7:**
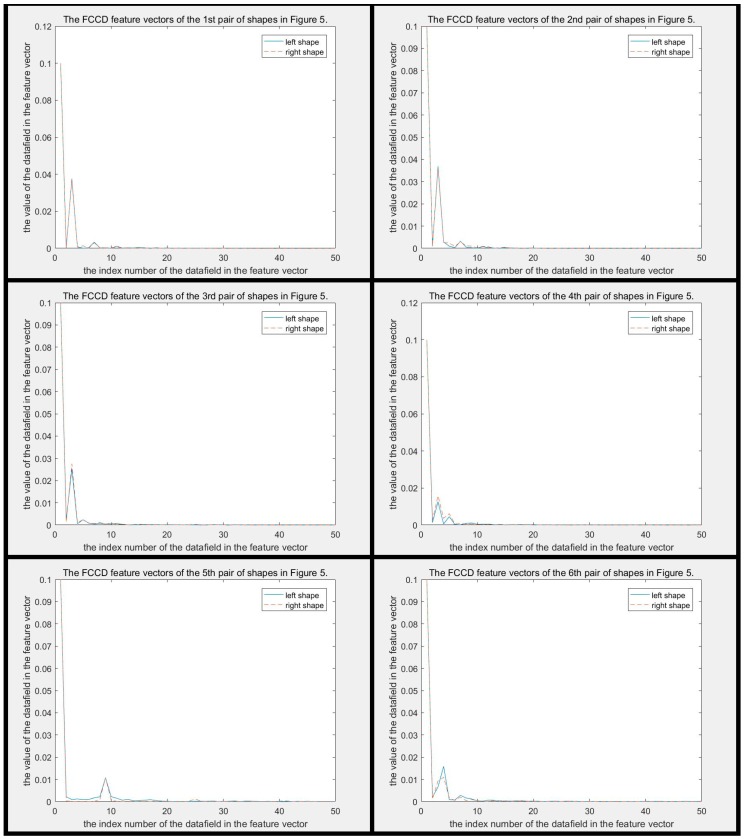
This figure shows the difference between two FCCD feature vectors of each pair of shapes in Figure 5. The subfigure of each pair is arranged in the same order as in Figure 5. It can be seen that two curves of each pair are so similar, and even overlap.

In addition, the CCD method still has a more serious problem in that it is too poor to describe the detail of the contours. In Figure 5, the distance between each pair of shapes is larger than 0.0277 and smaller than 0.0907, which are all smaller than $dis_{ccd}^{s}$. Therefore, the CCD method cannot identify the difference between each pair of shapes in Figure 5. Why cannot the CCD method distinguish? Because the differences between them are local, but the CCD method is more concerned with global features. The difference between each pair of CCD feature vectors is shown in Figure 6. It can be seen that two CCD feature vectors of each pair of shapes are similar globally, though they are in different classes. These small local differences cause two shapes to be completely in different classes, but unfortunately small local differences do not obviously increase the distance between two shapes in the CCD feature space. The difference between FCCD features of each pair of shapes is shown in Figure 7. The situation is similar to that in CCD. When the CCD method and Fourier transform are used in combination, the discriminability is not substantially improved, though the efficiency of FCCD is much higher than CCD.

The FMSCCD method (Fourier descriptor based on multiscale CCD) is proposed to solve the problem of CCD ignoring local differences and direction information. In order to facilitate the calculation, the number of sampling points of the contour in FMSCCD must make $N_{p}=2^{t_{0}}+1,t_{0}\in Z^{+}$ be satisfied. In the CCD method, a constant global centroid point is always used. However, in the FMSCCD method, a novel dynamic centroid point is used. Before the distance from a contour point to the dynamic centroid point is calculated, the dynamic centroid point is calculated with Equation (10).

(10) $$\begin{array}{l} p_{dc}^{h,i}=\frac{1}{2^{t_{0}-h}+1}\sum_{j=i-2^{t_{0}-h-1}}^{i+2^{t_{0}-h-1}}p_{j}=(\frac{1}{2^{t_{0}-h}+1}\sum_{j=i-2^{t_{0}-h-1}}^{i+2^{t_{0}-h-1}}x_{j},\frac{1}{2^{t_{0}-h}+1}\sum_{j=i-2^{t_{0}-h-1}}^{i+2^{t_{0}-h-1}}y_{j}),\\ i=1,2,\ldots ,N_{p},h=0,1,2,\ldots ,t_{0}-1 \end{array}$$

where h indicates the level of the scale from global to local. The larger the value of h in Equations (10)–(12), the finer the obtained feature. Then, with $p_{dc}^{h,i}=(x_{dc}^{h,i},y_{dc}^{h,i})$, the unnormalized distance to the dynamic centroid point from $p_{i}$ is calculated with Equation (11).

(11) $$d_{udc,}^{h,i}={\left({\left(x_{i}-x_{dc}^{h,i}\right)}^{2}+{\left(y_{i}-y_{dc}^{h,i}\right)}^{2}\right)}^{1/2},i=1,2,\ldots ,N_{p},h=0,1,2,\ldots ,t_{0}-1$$

Next, the same normalization method is used.

(12) $$d_{dc}^{h,i}=d_{udc}^{h,i}\sqrt{N_{p}}/\sum_{i=1}^{N_{p}}d_{uc}^{i},i=1,2,\ldots ,N_{p},h=0,1,2,\ldots ,t_{0}-1$$

The dynamic centroid point distance sequence is $D_{dc}^{h}=\{d_{dc}^{h,1},d_{dc}^{h,2},\ldots ,d_{dc}^{h,N_{p}}\}$. When h=0, $D_{ccd}$ and $D_{dc}^{h}$ are as the same. Combining $D_{dc}^{h}$ of different h forms the MSCCD (multiscale CCD) feature.

For the sake of efficient matching, it is convenient to generate a frequency domain feature $F_{dc}^{h}$ through Fourier transform with $D_{dc}^{h}$.

(13) $$F_{dc}^{h}(k)=\frac{1}{N_{p}}\left|\sum_{i=0}^{N_{p}-1}d_{dc}^{h,i}e^{\frac{-j2\pi ik}{N_{p}}}\right|,k=0,1,\ldots ,N_{p}-1,h=0,1,2,\ldots ,t_{0}-1$$

Combining $F_{dc}^{h}$ of different h forms the FMSCCD feature. When h is large, the relative location of the dynamic centroid point is easily disturbed by the noise on the contour. It makes the robustness of $D_{dc}^{h}$ and $F_{dc}^{h}$ decrease as h increases. Therefore, weighted summation is used when more than one scale is selected to form the multiscale features MSCCD and FMSCCD.

When a MSCCD feature is matched to another one, Equation (14) is used. When an FMSCCD feature is matched to another one, Equation (15) is used.

(14) $$\begin{array}{l} dis_{mc}(s_{1},s_{2})=\underset{0\leq n<N_{p}}{\min}\;(\sum_{h=0}^{H}\left(w_{h}\sum_{i=1}^{N_{p}}{\left(d_{msccd,s_{1}}^{h,i}-d_{msccd,s_{2}}^{h,n+i}\right)}^{2}\right))1/2,\\ n\in Z,0<H\leq t_{0}-1,H\in Z \end{array}$$

(15) $$\begin{array}{l} dis_{fmc}(s_{1},s_{2})=\sum_{h=0}^{H}\left(w_{h}\sum_{k=0}^{K}\left|F_{ccd}^{h,s_{1}}(k)-F_{ccd}^{h,s_{2}}(k)\right|\right),\\ K<N_{p},0<H\leq t_{0}-1,H\in Z \end{array}$$

where $w_{h}$ ($0<w_{h}\leq 1$) means the weight of the feature at the scale of level h. In this paper, $\alpha_{h}={\left(1-0.1h\right)}^{e_{w}}$, $w_{h}=\alpha_{h}/\sum_{i_{h}=0}^{H}\alpha_{i_{h}}$ and 0≤h<10 exist always. $w_{h}$ decreases as h increases with the fact the robustness becomes lower when h grows. H is the largest used value of h. In general, if the detail feature of a shape needs to be extracted, H should be larger, and if the global feature needs to be extracted, H should be smaller. The effect of $e_{w}$ and H to the discriminability of FMSCCD is shown in the experiments in Section 3.

**Figure 8:**
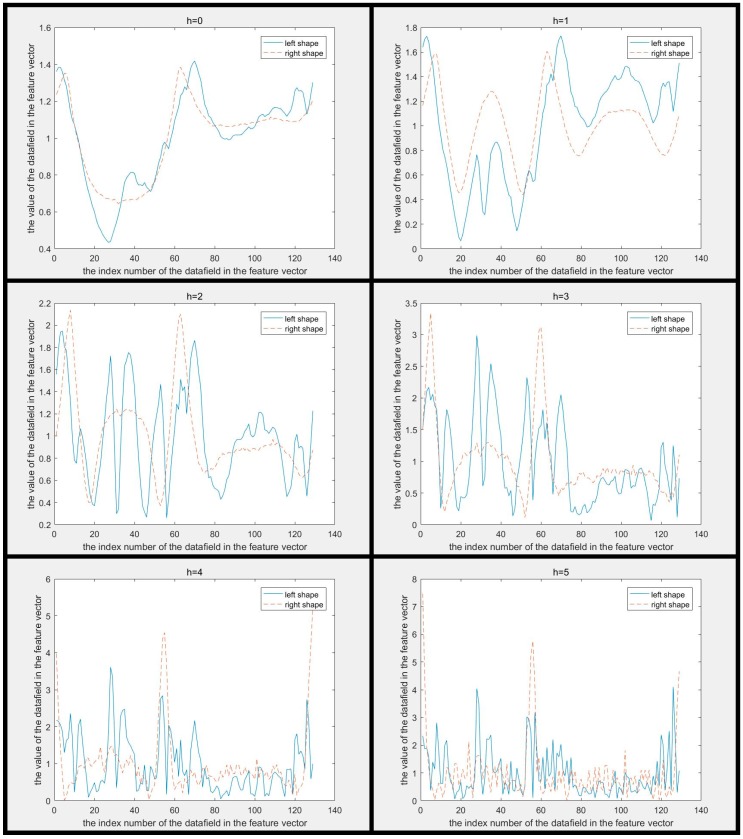
The difference between multiscale CCD (MSCCD) feature vectors of two shapes in Figure 2 at each scale (h=0,1,…,5). It can be seen that the difference between the two features becomes larger as h increases.

**Figure 9:**
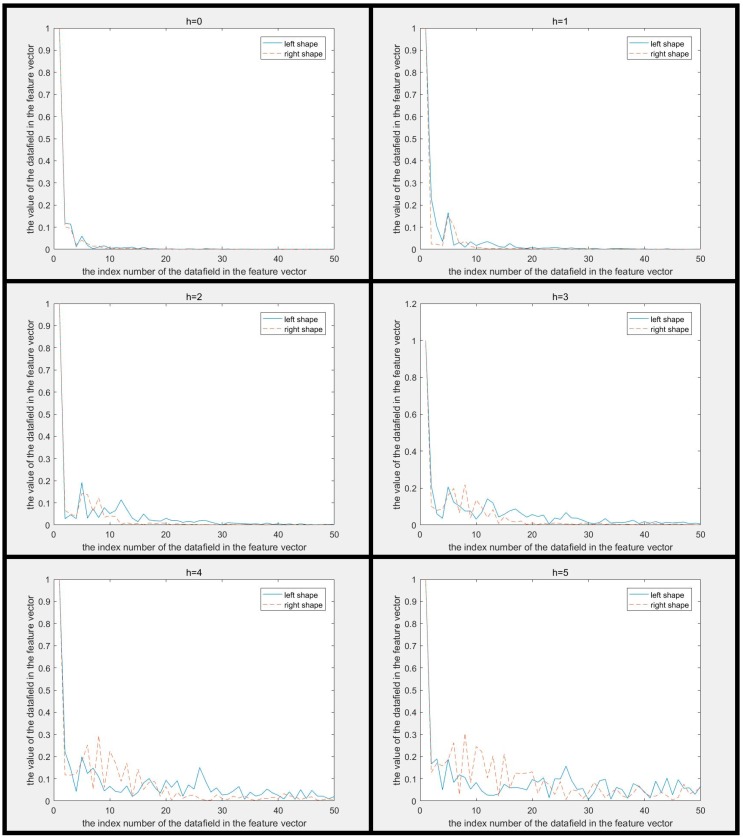
The difference between Fourier descriptor based on multiscale centroid contour distance (FMSCCD) feature vectors of two shapes in Figure 2 at each scale (h=0,1,…,5). It can be seen that the difference between the two features becomes larger as h increases.

The MSCCD method and the FMSCCD method are used to determine the differences between two shapes in Figure 2. The difference between MSCCD feature vectors of two shapes at each scale is shown in Figure 8. The difference between FMSCCD feature vectors of two shapes at each scale is shown in Figure 9. It can be seen that at some scales, the difference between features is larger than that in Figure 3 and Figure 4.

**Figure 10:**
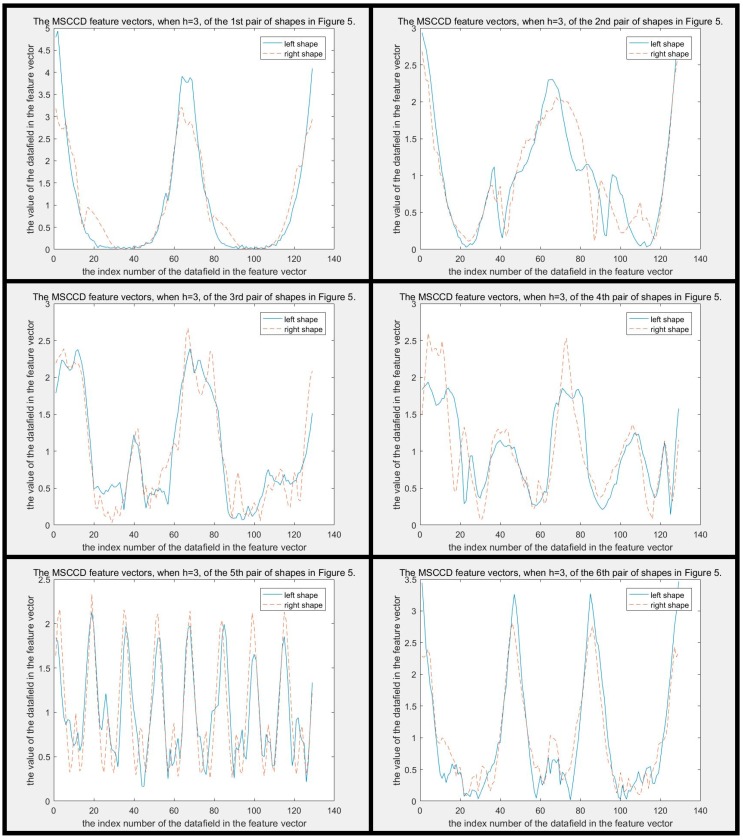
The difference between MSCCD feature vectors of each pair of shapes in Figure 5 when h=3. It can be seen that the difference between each pair of MSCCD features is larger than that in the corresponding subfigure in Figure 6.

**Figure 11:**
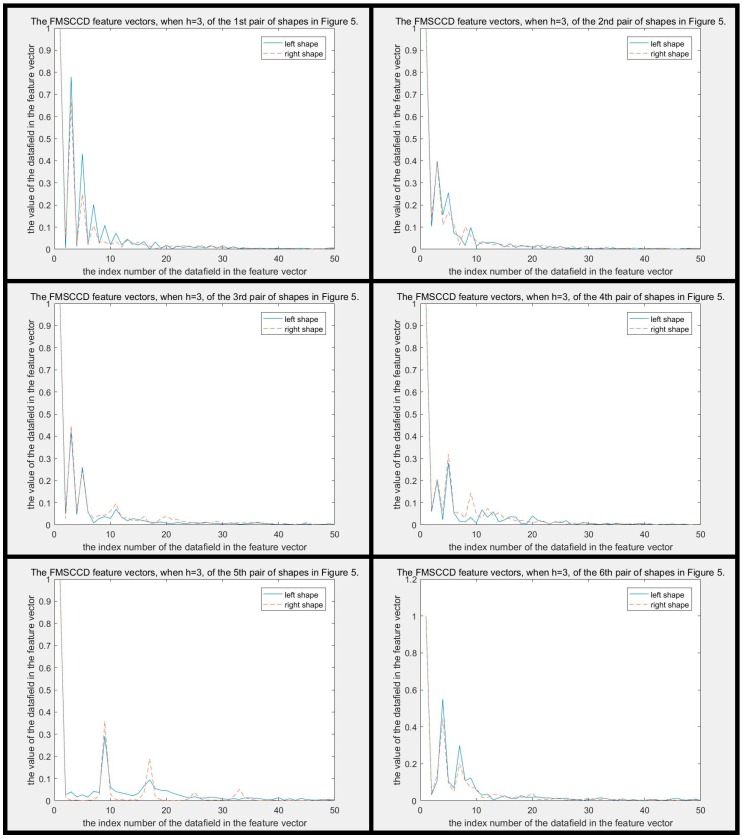
The difference between FMSCCD feature vectors of each pair of shapes in Figure 5 when h=3. It can be seen that the difference between each pair of FMSCCD features is larger than that in the corresponding subfigure in Figure 6.

The difference between MSCCD feature vectors of each pair of shapes in Figure 5 when h=3 is shown in Figure 10. The difference between FMSCCD feature vectors of each pair of shapes in Figure 5 when h=3 is shown in Figure 11. Clearly, the difference between each pair of shapes in Figure 10 and Figure 11 becomes larger compared to that in Figure 6 and Figure 7, respectively. This general improvement confirms the robustness of MSCCD and FMSCCD. The experiment results on three different databases in Section 3 prove the robustness of FMSCCD further.

FMSCCD (improved CCD) is generally used in combination with FASD (improved ASD) as CCD and ASD are complementary [18]. FMSCCD is also easy to use in combination with other features because the FMSCCD feature is easy to implement and has low computational cost during feature extracting and matching processes.

## 3. Results

In order to evaluate the performance of FMSCCD, CCD [18], FD-CCD [11], DIR [17], ASD&CCD [18], FPD (farthest point distance) [24], and MDM [16] were used for comparison. Since FMSCCD is a shape descriptor, the evaluation experiment is still on the well-known shape databases MPEG-7 CE1 Part B, Swedish Plant Leaf, and Kimia 99, on which the performance of other descriptors is reported. These algorithms were implemented in MATLAB, on a PC with I7 CPU, 16GB RAM under Windows 10 system. In all the experiments $N_{p}$ is 513.

In the experiments, when FMSCCD combined with other descriptors, the weighted distance was used to calculate the dissimilarity between two shapes with Equation (16).

(16) $$\begin{array}{l} dis_{wn}=w_{fmc}dis_{fmc}/\sum_{s_{2}=1}^{N_{d}}\sum_{s_{1}=1}^{N_{d}}dis_{fmc}(s_{1,}s_{2})+(1-w_{fmc})dis_{afs}/\sum_{s_{2}=1}^{N_{d}}\sum_{s_{1}=1}^{N_{d}}dis_{afs}(s_{1,}s_{2}),\\ 0<w_{fmc}<1 \end{array}$$

where $dis_{fmc}$ is the distance between two shapes in the FMSCCD feature space and $dis_{afs}$ is the distance between two shapes in another feature space (for example FASD, DIR, or MDM).

### 3.1. On MPEG-7 CE1 Part B

**Figure 12:**
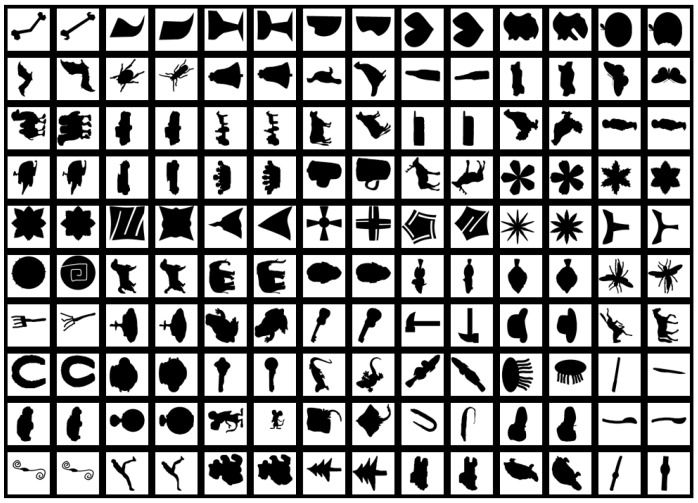
Some examples (a pair of shapes in each class) in MPEG-7 CE1 Part B.

MPEG-7 CE1 Part B is a common shape database used by a large number of shape descriptors in articles [8,10,11,16,17,18]. It contains 70 classes, each containing 20 shapes, so a total of 1400 shapes are in this database. Some examples in the database are shown in Figure 12.

“Bulls-eye-test” is a commonly used evaluation method [8,10,11,16,17,18] on this database. It is used to measure the performance of a descriptor. Each shape in the database is set as query in turn, then in the retrieval result corresponding to each query the number of correct hits (the retrieved shape and the query belong to the same class) of the top 40 most similar shapes to the query are counted. The counted number divided by 28,000 (the maximum of correct hits is 1400×20=28,000) is the bulls-eye-test score.

Matching time refers to the time taken to calculate the dissimilarity between the feature of query and the features of all shapes in the database. Matching time is used to evaluate the performance of the descriptor in terms of efficiency.

Table 1 shows the bulls-eye-test scores of FMSCCD when $e_{w}$ and H varies. It can be seen that when $e_{w}=5$, H=6,7 and 8, FMSCCD obtains the highest score of 75.73%. Table 2 shows the scores of FMSCCD+FASD when $w_{fms}$ varies with H=6 and $e_{w}=5$. It can be seen that when $w_{fms}=4/6$, FMSCCD+FASD obtains its highest score of 78.18%. In the remaining experiments H=6, $e_{w}=5$, and $w_{fms}=4/6$ are used without fine tuning to show the robustness of the proposed method.

The bulls-eye-test scores and matching time of some state-of-the-art descriptors and FMSCCD+FASD are shown in Table 3. It can be seen that the FMSCCD+FASD has the highest bulls-eye-test score (78.18%), among DIR [17] (77.69%), ASD&CCD [18] (76.20%), FASD (73.56%), MDM [16] (70.46%), FD-CCD [11] (67.94%), CCD [18] (68.67%), and FPD [24] (64.29%). In terms of efficiency, FPD (2.8 ms) [24] is the fastest, and FMSCCD+FASD (10.6 ms) is faster than ASD&CCD [18] (230.5 ms) and MDM [16] (30.2 ms). The experimental results show that FMSCCD+FASD has a strong discriminability and a great improvement in efficiency relative to ASD&CCD.

### 3.2. On Swedish Plant Leaf

**Figure 13:**
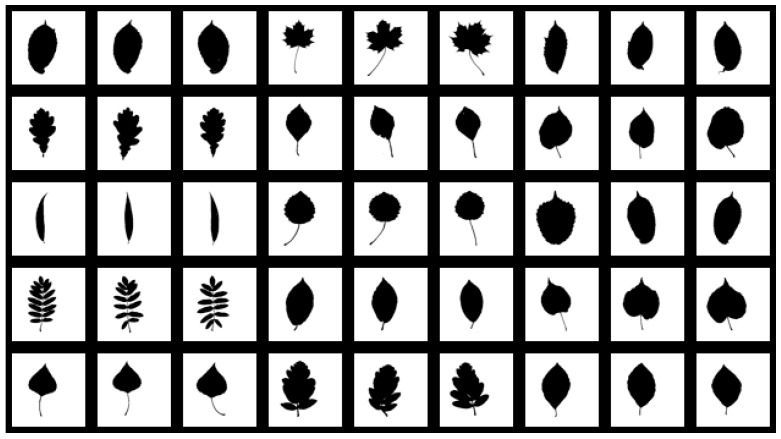
Some examples (three shapes in each class) in the Swedish Plant Leaf database.

As the FMSCCD is a shape descriptor, it is necessary to evaluate the performance on plant leaf retrieval, which is a common application for shape descriptors. Swedish Plant Leaf is a database of plant leaf images. It contains 15 classes, each containing 75 shapes, so a total of 1125 shapes are in the database. Some shapes in the database are shown in Figure 13.

Each shape in the database is set as a query in turn, then the similar shapes are retrieved in this database. In the retrieval results, the precision is calculated when 10 (recall rate is 13.33%), 20 (recall rate is 26.7%), 30 (recall rate is 40.0%), 40 (recall rate is 53.3%), 50 (recall rate is 66.7%), 60 (recall rate is 80.0%), 70 (recall rate is 93.3%), and 75 (recall rate is 100%) shapes are retrieved correctly [21]. The average precision is used to evaluate the performance of the proposed FMSCCD+FASD compared to some state-of-the-art methods. In terms of efficiency, the performance of each descriptor is independent of the specific database, so the matching time, which maintains the same trend as in MPEG-7 CE1 Part B, is no longer calculated.

In this experiment, the descriptor DALR (deep autoencoder learning representation) [21] based on the autoencoder is also selected to be compared. The experimental results of some state-of-the-art descriptors and the proposed FMSCCD in this paper are shown in Table 4. It can be seen that FMSCCD+FASD (68.3%) performs the best among DIR (67.6%), ASD&CCD (57.3%), MDM (54.6%), DALR (54.2%), and FD-CCD (49.0%). It can also be seen that in some scenarios, descriptors based on machine learning have no obvious advantages.

### 3.3. On Kimia 99

**Figure 14:**
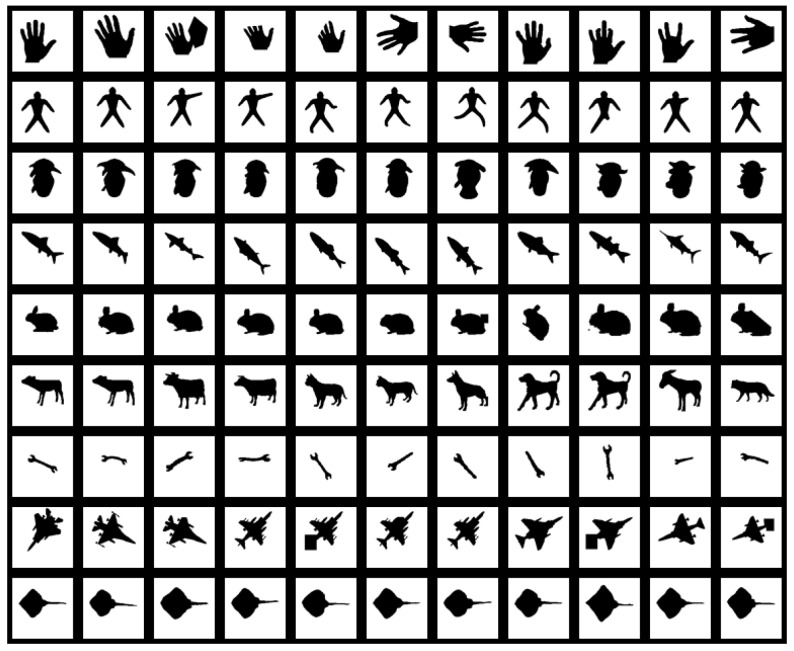
All shapes in the Kimia 99 database.

Kimia 99 is also a common shape database [8]. A large number of shape descriptors in their articles use Kimia 99 as a test database. It contains 9 classes, each containing 11 shapes, so a total of 99 shapes are in the database. All shapes in the database are shown in Figure 14.

Each shape in the database is set as a query in turn, then the similar shapes are retrieved in the remaining shapes. In the retrieval results, the numbers of correct hits from the first to the tenth most similar shapes of each query are counted. The final statistical results are used to evaluate the performance of the descriptors. The experimental results of some state-of-the-art descriptors and the proposed FMSCCD in this paper are shown in Table 5. It can be seen that the combination of FMSCCD and another descriptor always performs better than that descriptor alone. Experimental results show that FMSCCD is very flexible and performs well when combined with multiple descriptors.

## 4. Discussion

FMSCCD is a shape feature that is very simple in principle and structure and is easy to implement. The most important thing is that it has strong discriminability with low computational cost in feature extracting and matching processes, which is beneficial for engineering applications. In addition, because of its low computational cost, it can be easily combined with other features. As can be seen in experiments on MPEG-7 CE1 Part B, the FMSCCD performs better than other descriptors when FASD is used in combination. On the Swedish Plant Leaf database, FMSCCD+FASD performs best in average precision. In the experiment on the Kimia 99 database, the FMSCCD combined with multiple shape features performed better than a descriptor used alone.

## 5. Conclusions

FMSCCD is a simple, efficient, and compatible frequency domain descriptor. Multiscale description and Fourier transform are two useful tools for non-dynamic programming descriptors. A frequency domain descriptor can maintain high discriminability with low computational cost. The high discriminability in such highly efficient situations is what is needed for object recognition in remote sensing images. Another fact is that FMSCCD is also suitable for plant leaf retrieval.

## Figures and Tables

**Table 1:** Bulls-eye-test scores of FMSCCD when $e_{w}$ and H vary on MPEG-7 CE1 Part B.

	$e_{w}=1$	$e_{w}=2$	$e_{w}=3$	$e_{w}=4$	$e_{w}=5$	$e_{w}=6$	$e_{w}=7$	$e_{w}=8$
H=0	68.21%	68.21%	68.21%	68.21%	68.21%	68.21%	68.21%	68.21%
H=1	71.50%	71.65%	71.82%	71.93%	72.01%	71.98%	71.92%	71.83%
H=2	72.35%	72.74%	73.16%	73.48%	73.72%	73.80%	73.75%	73.76%
H=3	72.82%	73.52%	74.20%	74.56%	74.75%	74.91%	74.85%	74.81%
H=4	72.10%	73.45%	74.40%	75.15%	75.45%	75.44%	75.40%	75.17%
H=5	70.91%	73.09%	74.56%	75.25%	75.64%	75.60%	75.55%	75.28%
H=6	70.17%	72.91%	74.55%	75.32%	**75.73%**	75.70%	75.58%	75.28%
H=7	70.01%	72.86%	74.54%	75.34%	**75.73%**	75.71%	75.58%	75.29%
H=8	70.00%	72.87%	74.55%	75.36%	**75.73%**	75.71%	75.58%	75.29%

**Table 2:** Bulls-eye-test score of FMSCCD+ Fourier descriptor based on angle scale descriptor (FASD) when $w_{fms}$ varies with H=6.and $e_{w}=5$ on MPEG-7 CE1 Part B.

$w_{fms}$	1/6	2/6	3/6	4/6	5/6
Retrieval rate	76.83%	77.69%	77.89%	**78.18%**	77.80%

**Table 3:** Bulls-eye-test score and matching time of some descriptors on MPEG-7 CE1 Part B. DIR: distance interior ratio; ASD: angle scale descriptor; MDM: multiscale distance matrix; FD-CCD: Fourier descriptor based on CCD.

Method	Score	Matching Time (ms)
**FMSCCD + FASD (ours)**	**78.18%**	10.6
DIR [17]	77.69%	4.6
ASD&CCD [18]	76.20%	230.5
FASD	73.56%	5.6
MDM [16]	70.46%	30.2
FD-CCD [11]	67.94%	3.2
FPD [24]	64.29%	2.8
CCD [18]	68.67%	112.3

**Table 4:** The precision of some methods when recall varies on the Swedish Plant Leaf database.

Method	13.3%	26.7%	40.0%	53.3%	66.7%	80.0%	93.3%	100.0%	Average
**FMSCCD + FASD**	**92.7%**	**87.9%**	**83.2%**	**77.5%**	**70.4%**	**60.6%**	46.7%	27.6%	**68.3%**
DIR [17]	91.1%	86.5%	81.6%	75.2%	67.8%	59.4%	**47.4%**	**31.7%**	67.6%
ASD&CCD [18]	86.9%	79.9%	72.9%	64.6%	55.7%	44.5%	32.1%	21.8%	57.3%
MDM [16]	87.6%	78.8%	69.4%	60.9%	51.1%	41.7%	28.4%	18.7%	54.6%
DALR [21]	85.6%	74.6%	66.1%	58.3%	51.1%	42.4%	31.8%	23.9%	54.2%
FD-CCD [11]	78.4%	69.1%	61.4%	54.2%	46.4%	37.7%	27.1%	17.7%	49.0%

**Table 5:** Correct hits of some methods on top 10 most similar shapes on Kimia 99.

Method	1^st^	2^nd^	3^rd^	4^th^	5^th^	6^th^	7^th^	8^th^	9^th^	10^th^	Sum
FASD	95	88	81	74	63	57	63	54	47	38	660
FASD+FMSCCD	99	95	92	92	80	74	69	55	46	46	748
MDM [16]	97	94	92	83	78	80	61	69	55	51	760
MDM+FMSCCD	97	95	94	88	89	80	73	64	61	54	795
DIR [17]	97	92	88	84	89	79	84	76	71	58	818
**DIR+FMSCCD**	99	94	94	93	91	86	84	83	73	58	**855**

## References

[1] Alajlan N., El Rube I., Kamel M.S., Freeman G. (2007). Shape retrieval using triangle-area representation and dynamic space warping. Pattern Recognit..

[2] Zahn C.T., Roskies R.Z. (1972). Fourier descriptors for plane closed curves. IEEE Trans. Comput..

[3] Wang B., Gao Y., Sun C., Blumenstein M., La Salle J. Can walking and measuring along chord bunches better describe leaf shapes?. Proceedings of the IEEE Conference on Computer Vision and Pattern Recognition.

[4] Felzenszwalb P.F., Schwartz J.D. Hierarchical matching of deformable shapes. Proceedings of the IEEE Conference on Computer Vision and Pattern Recognition.

[5] Hu R.-X., Jia W., Zhao Y., Gui J. (2012). Perceptually motivated morphological strategies for shape retrieval. Pattern Recognit..

[6] Premachandran V., Kakarala R. (2013). Perceptually motivated shape context which uses shape interiors. Pattern Recognit..

[7] Yang X., Bai X., Latecki L.J., Tu Z. Improving shape retrieval by learning graph transduction. Proceedings of the European Conference on Computer Vision.

[8] Ling H., Jacobs D.W. (2007). Shape classification using the inner-distance. IEEE Trans. Pattern Anal. Mach. Intell..

[9] Ling H., Jacobs D.W. Using the inner-distance for classification of articulated shapes. Proceedings of the IEEE Computer Society Conference on Computer Vision and Pattern Recognition.

[10] Belongie S., Malik J., Puzicha J. (2002). Shape matching and object recognition using shape contexts. IEEE Trans. Pattern Anal. Mach. Intell..

[11] Zhang D., Lu G. (2005). Study and evaluation of different fourier methods for image retrieval. Image Vis. Comput..

[12] Yang H.S., Lee S.U., Lee K.M. (1998). Recognition of 2d object contours using starting-point-independent wavelet coefficient matching. J. Vis. Commun. Image Represent..

[13] Bai X., Wang B., Yao C., Liu W., Tu Z. (2012). Co-transduction for shape retrieval. IEEE Trans. Image Process..

[14] Yang X., Koknar-Tezel S., Latecki L.J. Locally constrained diffusion process on locally densified distance spaces with applications to shape retrieval. Proceedings of the IEEE Conference on Computer Vision and Pattern Recognition.

[15] Kontschieder P., Donoser M., Bischof H. Beyond pairwise shape similarity analysis. Proceedings of the Asian Conference on Computer Vision.

[16] Hu R.X., Jia W., Ling H., Huang D. (2012). Multiscale distance matrix for fast plant leaf recognition. IEEE Trans. Image Process..

[17] Kaothanthong N., Chun J., Tokuyama T. (2016). Distance interior ratio: A new shape signature for 2d shape retrieval. Pattern Recognit. Lett..

[18] Fotopoulou F., Economou G. Multivariate angle scale descriptor of shape retrieval. Proceedings of the SPAMEC.

[19] Xie J., Heng P.A. (2008). Shape matching and modeling using skeletal context. Pattern Recognit..

[20] Amor B.B., Su J., Srivastava A. (2016). Action recognition using rate-invariant analysis of skeletal shape trajectories. IEEE Trans. Pattern Anal. Mach. Intell..

[21] Xu G., Fang W. Shape retrieval using deep autoencoder learning representation. Proceedings of the International Computer Conference on Wavelet Active Media Technology & Information Processing.

[22] Mohanty N., Rath T., Lee A., Manmatha R. Learning shapes for image classification and retrieval. Proceedings of the International Conference on Image & Video Retrieval.

[23] Conoci S., Rundo F., Petralta S., Battiato S. Advanced skin lesion discrimination pipeline for early melanoma cancer diagnosis towards PoC devices. Proceedings of the European Conference on Circuit Theory & Design.

[24] El-ghazal A., Basir O., Belkasim S. (2009). Farthest point distance: A new shape signature for fourier descriptors. Signal Process. Image Commun..

